# Ibrutinib in Refractory or Relapsing Primary Central Nervous System Lymphoma: A Systematic Review

**DOI:** 10.3390/neurolint14010009

**Published:** 2022-01-11

**Authors:** Gaurav Nepal, Mahika Khurana, Domenica Herrera Bucheli, Siddhartha Bhandari, Utsav Joshi, Riwaj Bhagat, Jessica Holly Rehrig, Prasun Pudasainee, Yow Ka Shing, Juan Fernando Ortiz, Rajeev Ojha, Bikram Prasad Gajurel, Jonathan Quinonez, Samir Ruxmohan, Trevine Albert, Steven Licata, Joel Stien

**Affiliations:** 1 Department of Internal Medicine, Tribhuvan University Institute of Medicine, Maharajgunj, Kathmandu 44600, Nepal; gauravnepal@iom.edu.np (G.N.); siddhartb123@gmail.com (S.B.); 2 Department of Public Health, Berkeley Public Health Division, Berkeley, CA 94704, USA; mahika_khurana@berkeley.edu; 3 Department of Medicine, Universidad Internacional de Ecuador, Loja 110150, Ecuador; doherrerabu@gmail.com; 4 Department of Internal Medicine, Rochester General Hospital, Rochester, NY 14621, USA; utsavjoshi.uh@gmail.com; 5 Department of Neurology, Boston University Medical Center, Boston, MA 02118, USA; forriwaj@gmail.com; 6 Department of Neurology, North Shore University Hospital, Manhasset, NY 11030, USA; jrehrig@northwell.edu; 7 Department of Internal Medicine, AMITA Health Saint Francis Hospital, Evanston, IL 60202, USA; ppudasainee1@gmail.com; 8 Department of Internal Medicine, National University Hospital, Singapore 119074, Singapore; yow_ks@yahoo.com.sg; 9 Department of Neurology, California Institute of Behavioral Neuroscience & Psychology, Fairfield, CA 94534, USA; sumjuanfer41@gmail.com; 10 Department of Neurology, Tribhuvan University Teaching Hospital, Kathmandu 44600, Nepal; rajeevnet@hotmail.com (R.O.); bikram_gajurel@hotmail.com (B.P.G.); 11 Department of Neurology, Larkin Community Hospital, South Miami, FL 100181, USA; sruxmohan@larkinhospital.com; 12 Department of Osteopathic Neuromuscular Medicine, Larkin Community Hospital, Miami, FL 100181, USA; trevine1@gmail.com (T.A.); drslicata@gmail.com (S.L.); ommdoc@aol.com (J.S.)

**Keywords:** ibrutinib, BTK, Bruton tyrosine kinase inhibitor, PCNSL, primary central nervous system lymphoma, lymphoma

## Abstract

Primary Central Nervous System Lymphoma (PCNSL) is a rare variant of Non-Hodgkin Lymphoma (NHL) representing 1–2% of all NHL cases. PCNSL is defined as a lymphoma that occurs in the brain, spinal cord, leptomeninges, or eyes. Efforts to treat PCNSL by traditional chemotherapy and radiotherapy have generally been unsuccessful as a significant proportion of patients have frequent relapses or are refractory to treatment. The prognosis of patients with Refractory or Relapsed (R/R) PCNSL is abysmal. The optimal treatment for R/R PCNSL is poorly defined as there are only a limited number of studies in this setting. Several studies have recently shown that ibrutinib, a Bruton tyrosine kinase (BTK) inhibitor, has promising results in the treatment of R/R PCNSL. However, these are preliminary studies with a limited sample size. In this systematic review, we explored and critically appraised the evidence about the efficacy of the novel agent ibrutinib in treating R/R PCNSL.

## 1. Introduction

PCNSL is a rare variant of NHL representing 1–2% of all NHL cases. PCNSL is defined as a lymphoma that presents in the brain, spinal cord, leptomeninges, or eyes, restricted entirely to the craniospinal axis [1]. Close to 90% of cases are Diffuse Large B Cell Lymphoma (DLBCL), with the large majority of the remaining being lymphoblastic B-cell lymphoma, Burkitt’s and Burkitt-like lymphoma, as well as T-cell lymphoma [2,3].

For a long time, efforts to treat PCNSL with systemic regimes used in DLBCL have generally been unsuccessful. Whole-Brain Radiation Therapy (WBRT) was once the standard of care for PCNSL. However, the appearance of severe neurotoxicity in surviving patients, including delayed neurotoxicity, poor long-term survival, and low quality of life, resulted in its downfall [4]. Standard chemotherapy for systemic NHL, such as Cyclophosphamide, Doxorubicin, Vincristine, Prednisone (CHOP)-based regimens, were also ineffective for PCNSL, likely due in part to the low penetration of the blood–brain barrier (BBB) [5]. With the introduction of High-Dose Methotrexate (HD-MTX) in the first-line treatment of PCNSL, survival outcomes have greatly improved. Unlike other chemotherapeutic agents, HD-MTX is given directly to the subarachnoid and ventricular space. It has been shown to prolong overall survival and currently forms the backbone of PCNSL therapy [6]. However, despite high response rates with initial HD-MTX-based treatment, more than half of the initial responders relapse. Moreover, about 25% of patients have a disease that fails to respond to initial treatment. The prognosis of Refractory or Relapsed (R/R) PCNSL is grim, and the optimal treatment is poorly elucidated as there have only been a limited number of studies conducted in this setting [7].

**Figure 1:**
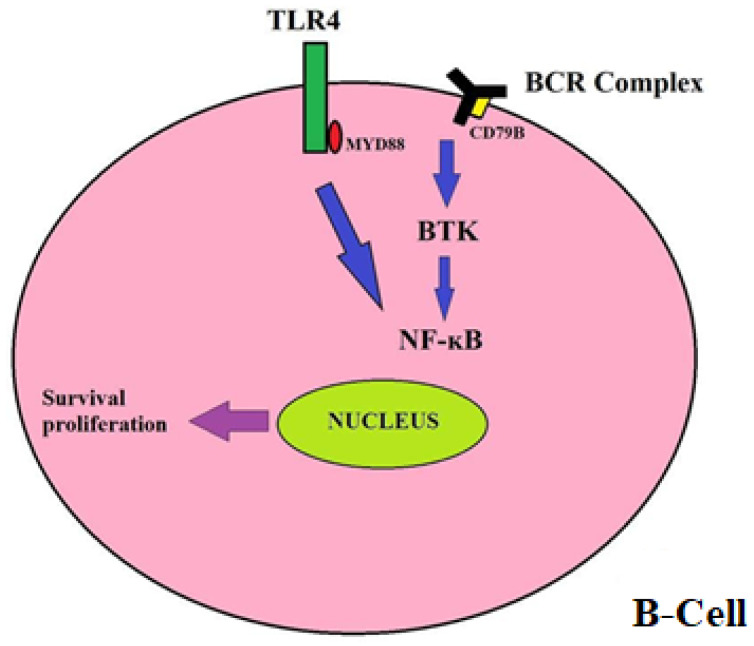
In normal B-cells, Toll-Like Receptor (TLR) signaling cooperates with B-Cell Receptor (BCR) signaling to activate pro-survival transcription factor, Nuclear Factor-κB (NF-ĸB). Mutation in the CD79B protein (a subunit of the B-cell receptor) and MYD88 protein (adaptor protein of the Toll-like receptor) activates NF-κB signaling and subsequently promotes the survival and proliferation of B-cells. Abbreviations: BCR, B-Cell Receptor; TLR4, Toll-Like Receptor 4; BTK, Bruton Tyrosine Kinase; NF-κB, Nuclear Factor-κB.

To achieve better treatment strategies, genomic studies have been conducted to find driver mutations behind PCNSL. A total of 37 different gene mutations are involved in the pathophysiology of PCNSL [8], the most important being a gain of function mutation in the CD79B protein (a subunit of the B-cell receptor) and MYD88 protein (adaptor protein of the Toll-like receptor) [9]. In normal B-cells, Toll-Like Receptor (TLR) signaling cooperates with B-Cell Receptor (BCR) signaling to activate the pro-survival transcription factor Nuclear Factor-κB (NF-ĸB) (Figure 1). Mutated MYD88 protein and mutated CD79B protein activate NF-κB signaling and subsequently promote the survival and proliferation of B-cells [10,11]. This signaling pathway is mediated downstream by Bruton Tyrosine Kinase (BTK).

Thus, there was much excitement when ibrutinib was discovered, an irreversible inhibitor of BTK, with good CSF penetration. Naturally, it was thought to be a promising agent for R/R PCNSL, interfering with B-cell proliferation mediators in the CNS [12]. Ibrutinib was FDA approved for mantle cell lymphoma in 2013, which then expanded its horizon for chronic lymphocytic leukemia, small lymphoma, Waldenström’s macroglobulinemia, and marginal zone lymphoma [13]. In this systematic review, we explore the evidence about the efficacy of the novel agent ibrutinib to treat R/R PCNSL.

## 2. Materials and Methods

Our systematic review explored the efficacy and safety of ibrutinib in R/R PCNSL according to the Preferred Reporting Items for Systematic Review and Meta-Analysis (PRISMA) statement in conjunction with the PRISMA checklist and flow diagram [14].

Literature search and selection:

A comprehensive electronic literature search was performed on PubMed, Google Scholar, Embase, Cochrane database, and CNKI for the published English-language articles from 1 January 2005 to 1 July 2020. Searches were conducted using the keywords “ibrutinib” or “Bruton tyrosine kinase inhibitor” in combination with “PCNSL”, “primary central nervous lymphoma”, “CNS lymphoma”, or “lymphoma”. Boolean logic was used for conducting the database search, and the Boolean search operators “AND” and “OR” were used to link search terms. For the advanced PubMed search, the Medical Subject Headings (MeSH) database was used to find MeSH terms for the aforementioned search terms. Boolean logic and the Boolean search operators “AND” and “OR” were used to link search terms in the PubMed search. For each study shortlisted via this process, the paper’s reference section was checked to identify further studies not found in previous database searches.

Furthermore, repositories of grey literature and preprint servers were also searched. All studies obtained from the methods as mentioned earlier were imported to Mendeley library. A check for duplicates was run on Mendeley, which displayed a list of duplicate references, which were then removed. After that, studies were screened manually for duplicates by two independent reviewers (G.N. and R.O.). The titles and abstracts of the studies remaining after duplicate removal were screened independently by G.N. and R.O. Potentially relevant full texts were then screened based on the inclusion and exclusion criteria listed below. Any discrepancies were resolved through a discussion with a third reviewer, S.B. For instances where multiple publications of the same data existed, the most comprehensive and up-to-date source was selected over the rest.

Inclusion criteria:

- Study type(s): clinical trials, prospective, or retrospective studies published in the English language were considered eligible for this study;
- Study participant(s): patients of any age with R/R PCNSL, irrespective of subtypes, treated with ibrutinib monotherapy or combination therapy;
- Study outcome(s): reporting either efficacy or safety endpoints, including the Overall Response (ORR), Complete Response (CR), Partial Response (PR), Progression-Free Survival (PFS), Overall Survival (OS), and adverse events.

Exclusion criteria:

- Case reports and case series with ≤ 2 cases;
- Review articles;
- Studies that were published in a language other than English;
- Research that did not report the outcomes of interest listed above.

Data extraction: 

The final included studies were collated, and the two reviewers (G.N. and R.O.) used standardized data extraction formats to extract the data. After extraction, both reviewers matched their data with each other and revisited papers where disagreements arose. Any discrepancies were resolved through a discussion with the third reviewer (S.B.). The following data were extracted from the included studies: authors, year of publication, country, study design, therapeutic regimen, follow-up period, number of patients, age, safety outcomes, and adverse events.

## 3. Results

**Figure 2:**
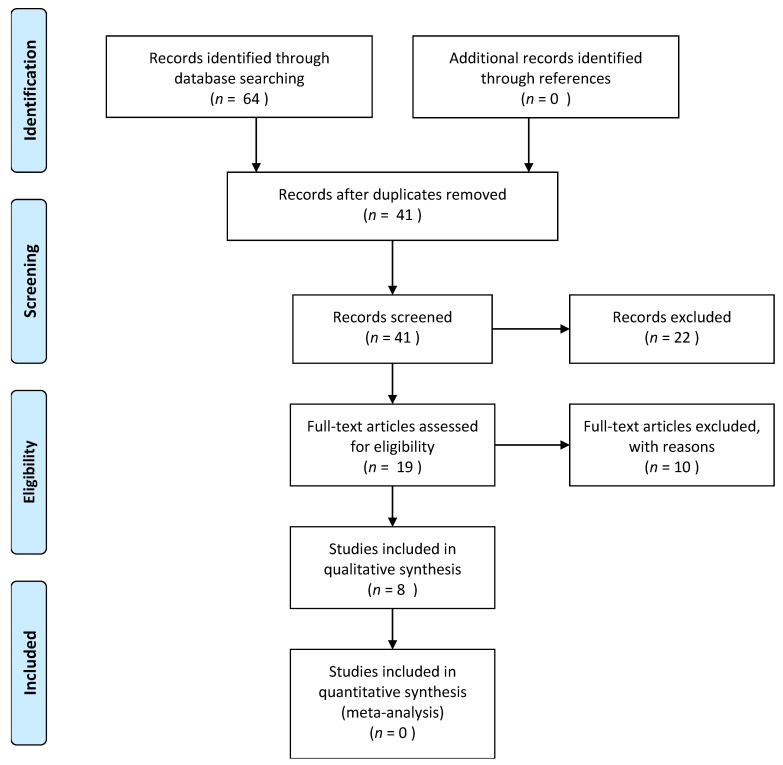
PRISMA flow diagram depicting the flow of information through the different phases of the systematic review.

Characteristics of included studies: 

In our systematic review, we included a total of eight studies evaluating the effects of ibrutinib for the treatment of R/R PCNSL. Figure 2 displays the results of our literature search and selection. Three studies originated from the USA [9,15,16]; one was conducted both in Belgium and France [17]; there was one each from Germany [18], Australia [19], France [20], and China [21]. The mean age of the patient, in all included studies, was above 60 y. The number of R/R PCNSL patients recruited in the included studies ranged from 3–52. Three studies were phase I clinical trials [9,15,16]. Patients in these studies were previously treated with HD-MTX with or without radiotherapy and received ibrutinib-based monotherapy, or combination therapy, or both in sequence. Ibrutinib was administered at a dose ranging from 560–840 mg. Four included studies adopted a retrospective design [17,18,19,21], analyzing patients heavily pretreated with HD-MTX with or without radiotherapy. The majority of patients in the retrospective studies received a 560 mg monotherapy of ibrutinib, with a small proportion receiving ibrutinib-based combination chemotherapy. A phase II clinical trial [20] evaluated 52 elderly patients who previously received HD-MTX chemotherapy and later received 560 mg of ibrutinib once daily as a monotherapy. The details of the included studies are tabulated in Table 1.

Response rate with Ibrutinib:

Studies used ibrutinib as a part of induction or consolidation monotherapy, a part of multidrug therapy, or radiotherapy to treat R/R PCNSL. Ibrutinib has shown promising results in all of the above approaches.

### 3.1. Monotherapy

In a retrospective study by Chamoun et al., 14 patients with relapsed or refractory PCNSL were treated with ibrutinib monotherapy. All patients had received previous HD-MTX chemotherapy. The overall response rate was 50% (7/14). Complete Response (CR) was observed in 21% (3/14) and Partial Response (PR) in 29% (4/14), and the median Progression-Free Survival (PFS) was 6 mo [17].

Soussain et al. included 52 patients with R/R PCNSL or primary vitreoretinal lymphoma of the DLBCL subtype in their phase II trial and administered ibrutinib monotherapy for them. All patients had previously been treated with HD-MTX, and seven had additionally received autologous stem cell transplantation. After 2 mo of treatment, 44 patients remained in the study for analysis. Among them, 70% of patients achieved disease control (31/44), 23% achieved CR or Complete Response unconfirmed (CRu) (10/44), 36% achieved PR (16/44), and 11% had stable disease (5/44). The overall response rate, including CR, CRu, and PR, was 60% (31/52). In the intention to treat analysis (*n* = 52), the disease control and the Overall Response Rate (ORR) after 2 mo of treatment were 62% and 52%, respectively [20]. 

In a phase I study by Grommes et al., 13 patients with R/R PCNSL were included and treated with ibrutinib monotherapy. Twelve of thirteen patients with PCNSL were evaluated for response, as one patient was not evaluable because the drug was discontinued within 14 d of treatment due to personal choice. There were 77% (10/13) of patients showing a clinical response, including five patients with a complete response and five patients with a partial response. One additional patient experienced tumor regression; however, this did not meet the criteria for PR. The median PFS was 4.6 mo, and the median Overall Survival (OS) was 15 mo [15].

### 3.2. Combination Therapy

In another phase I study by Grommes et al., fifteen eligible patients were involved, among whom nine had PCNSL. There were 67% CR (6/9), 22% PR (2/9), and 11% (1/9) progressive disease in R/R PCNSL patients taking ibrutinib combination therapy. The overall response rate was 89% (8/9). The median PFS for all 15 patients was 9.2 mo. The median OS of all patients was not reached (11/15 subjects alive). The median PFS for the PCNSL patients was not reached at the time of publication. The 1 y OS for all patients was 71.1% [16].

In a phase Ib study by Lionakis et al., eighteen PCNSL patients were recruited, among which five were untreated patients, two were relapsed cases, and eleven were refractory to initial treatment. All 18 patients received the ibrutinib monotherapy window, followed by combination therapy. Among the 18 patients in the ibrutinib window study, 94% (17/18) had disease reduction, 83% (15/18) achieved PR, and none had CR. Out of 14 evaluable patients who underwent Dose-Adjusted Temozolomide, Etoposide, Doxil, Dexamethasone, Ibrutinib, and Rituximab (DA-TEDDi-R), 86% (12/14) achieved CR or CRu, and one achieved PR. Among all 13 patients with R/R disease, DA-TEDDi-R produced a median PFS of 15.3 mo. Sub-analysis of all eleven patients with refractory disease revealed a median PFS of 11.2 mo (95% CI 0.8 to undefined). Among the patients with R/R disease, the ORR was 53.8% (7/13), while 46% (6/13) progressed and/or died [9]. 

In a retrospective study by Lauer et al., both R/R PCNSL and isolated secondary CNS lymphoma patients were included. These patients were treated with either ibrutinib monotherapy or combination therapy. The ORR in R/R PCNSL was 60% (3/5) and 75% (3/4) in isolated secondary CNS lymphoma [18]. 

In another retrospective study by Lewis et al., 16 patients received ibrutinib either as a monotherapy or combination therapy. Half had R/R PCNSL, while the remaining half had R/R secondary CNS lymphoma. Among all patients, the ORR was 69%, with a CR rate of 63%. The ORR in PCNSL patients was 50% (*n* = 4) and in SCNSL patients was 88% (*n* = 7). With a median follow-up of 14 mo, calculated using the median observation period among patients alive at the last follow-up, the median PFS and OS were not reached. The 1 y PFS was 56% for the entire cohort, 50% for PCNSL, and 60% for SCNSL. The 1 y OS was 66% for the entire cohort, 50% for PCNSL, and 80% for SCNSL [19]. 

Mao et al. retrospectively reviewed the clinical data of 91 primary PCNSL patients. Among those who had R/R disease, ibrutinib was used in three R/R patients. All three patients received a wide array of treatment after being non-responsive to HD-MTX or having a relapse. However, ultimately, ibrutinib was used in all and had an ORR of 100% [21]. 

Adverse effects:

It is well known that ibrutinib is associated with immunosuppression and hematological toxicity, including lymphopenia, anemia, thrombocytopenia, and accompanying opportunistic infections such as Aspergillosis [22,23,24]. In the study by Lionakis et al., in which ibrutinib monotherapy induction was followed by combination chemotherapy, 39% (7/18) of patients developed pulmonary and cerebral Aspergillosis. Out of these seven patients, 29% (2/7) developed Aspergillosis during the ibrutinib monotherapy phase, while the other 71% (5/7) were detected after the DA-TEDDi-R regimen was initiated [9]. The rate of Aspergillosis was variable. The aspergillosis rates in Lionakis et al.’s study (39%; 7/18) [9] were much higher than those observed in Soussain et al.’s study (5%; 2/44) [20] and Grommes et al.’s study (7.7%; 1/13) [15]. Half of the included studies observed no fungal infection at all [16,17,19,21]. Additionally, altered liver function tests, hyperglycemia, and electrolyte imbalance were also reported as common adverse effects in most studies.

## 4. Discussion

Our systematic review showed that ibrutinib has promising results, either as a monotherapy or combination therapy. Expectedly, the rates of CR and PR were higher in combination therapy compared to monotherapy. The radiographic response of R/R PCNSL was higher with combination therapy compared to ibrutinib monotherapy [16], with more prolonged progression-free survival. The combination of radiotherapy with ibrutinib [18,19] and administering DA-TEDDI-R after ibrutinib both favored progression from PR to CR [9], most of which were durable with no further relapses [9]. Despite this, rituximab’s role as a combination with ibrutinib in the treatment of PCNSL has become questionable recently given its low BBB transition [21], with no significant difference being observed with or without rituximab in a recent trial [16].

Multiple mutations have been described in the pathogenesis of PCNSL, among which the mutations activating NF-κB appear to be the critical offender [8]. Combination therapy covers a wide range of such offending pathways, rendering synergistic effects and potentially greater efficacy than monotherapy at the expense of potentially more significant toxicity. In vitro cell line models have shown that DNA-damaging agents such as doxorubicin, etoposide, cytarabine, and mitomycin C have super-additive and/or synergistic efficacy in inhibiting the NF-κB signaling pathway. Anti-folate agents such as methotrexate, pyrimethamine, pralatrexate, and 4-aminofolic acid, on the other hand, display little if any synergistic effect with ibrutinib [9]. This finding is crucial; the antagonism between ibrutinib and multiple structurally distinct anti-folates indicates a class effect and suggests that ibrutinib might not improve MTX-based regimens’ efficacy, which is the standard treatment for PCNSL.

Interestingly, tumors without any mutations in the BCR pathway also showed CR to ibrutinib therapy [9,16]. Patients with mutations that might be expected to restore BCR pathway activity in the presence of ibrutinib also showed a response [16]. The gene mutation in the BCR pathway was found to have no direct implications on the tumor response rate [20], suggesting a potential alternative mechanism for ibrutinib’s action. Grommes et al. showed that the genomic alterations associated with the tumor were cleared after therapy. However, in relapse cases, these alterations recurred even before conventional CSF studies showed disease recurrence [16]. This implies that such genomic alterations may potentially be used as an early marker indicating disease relapse. Detection of such genomic alterations by analyzing circulating tumor DNA has been used to detect relapse in melanoma, breast cancer, and lung cancer [25,26,27]. 

Our review showed that Aspergillosis was clinically the most significant adverse effect of ibrutinib therapy. The rate of Aspergillosis was highly variable, ranging from none to 39%. Close observation of patients under treatment and rational use of antifungal treatment and prophylaxis are warranted given the number of cases seen. The pathophysiology behind Aspergillosis infection with the use of ibrutinib seems to lie in the innate immune system. B-cells do not play a significant role in antifungal immunity; hence, blockade of B-cell receptor signaling by ibrutinib is likely unrelated to Aspergillosis [28]. Instead, ibrutinib targets phagocytizing cells such as neutrophils and macrophages, which express BTK [29]. TLRs, NLRP3, TREM-1, and Dectin-1 can activate monocytes, macrophages, and neutrophils, and ibrutinib can block this activation through BTK-dependent processes. Ibrutinib can also inhibits T-cell differentiation, effector function, and survival by inhibiting IL-2-inducible T-cell Kinase (ITK) on T-cells [30]. A recent in vitro study has also demonstrated that ibrutinib-associated BTK depletion impairs NFAT and NF-κB responses in macrophages, leading to impaired clearance of *Aspergillus fumigatus* [31,32]. 

It is unknown whether second-generation BTK inhibitors such as tirabrutinib and acalabrutinib will outperform ibrutinib in the future [33]. Tirabrutinib was recently tested in Japan in a phase I/II dose escalation trial for the treatment of R/R PCNSL, and it showed promising results. Despite the fact that the PFS was just 2.9 mo, the overall response rate (ORR) was 64%. Tirabrutinib is highly selective for BTK, reducing toxicity in theory. Despite this, nearly half of the patients (47.7%) had an adverse event of grade 3 or above, with three cases of grade 3 skin reaction and one case of grade 5 interstitial lung disease with associated Pneumocystis jirovecii pneumonia [34]. In the United States, a phase II trial of tirabrutinib is expected (NCT04947319). Another second-generation BTK inhibitor, acalabrutinib, is now being tested in patients with R/R PCNSL (NCT04548648, NCT04462328).

Although our systematic review showed ibrutinib’s effectiveness in treating R/R PCNSL, our review has several limitations. In this review, the studies taken into consideration are all preliminary studies with limited sample sizes and are heterogeneous in terms of methodology, treatment regimens, and outcome variables, thereby limiting the generalization of the results. Additionally, these studies have mainly been performed on the elderly population. Pediatric PCNSL, though rare, warrants similar studies that could trial and establish a proper treatment regimen for its management. 

## 5. Conclusions

The irreversible BTK inhibitor ibrutinib has a promising effect on the treatment of R/R PCNSL, primarily when used in combination therapy. However, caution has to be taken with the choice of combination therapy because drugs such as anti-folate agents may have an antagonistic effect. Although ibrutinib is generally well tolerated, immunosuppression with opportunistic invasive Aspergillosis is frequent, particularly in combination therapy. Our findings provide preliminary evidence for the use of ibrutinib in patients with R/R PCNSL and highlight the necessity for a large multicenter prospective study and phase III randomized controlled clinical trials to thoroughly characterize the efficacy and safety of ibrutinib compared to existing therapies.

## Figures and Tables

**Table 1:** Key methodological characteristics of the included studies.

Study	Origin	Design	R/R Cases (*n*)	Median Age	Previous Treatment	Mono/Combined	Ibrutinib Dose	Median Follow-Up
Chamoun 2016	France and Belgium	Retrospective study	14	68 y (range 48–79)	High-dose methotrexate-based chemotherapy. The median number of previous therapies was 3 (range 2–9).	Monotherapy Four patients received steroids for cerebral edema	560 mg once daily 1 patient received a 420 mg dose	N/A
Lionakis 2017	USA	Phase I clinical trial	13	66 (range 49–87)	Median of 2 (range 1–6) prior treatments	Monotherapy window followed by DA-TEDDi-R combination therapy	560–840 mg	N/A
Grommes 2017	USA	Phase I clinical trial	13	69 (60–80)	All received high-dose methotrexate-based chemotherapy. Two received radiation. Median 2 (1–8) before treatment	Monotherapy	560 and 840 mg	479 days (range, 354–739)
Grommes 2019	USA	Phase I clinical trial	9	62 y (range, 23–74)	High-dose methotrexate-based chemotherapy with a heterogeneous combination of rituximab, an alkylating agent, radiation therapy, and stem cell therapy	Ibrutinib-based combination therapy followed by ibrutinib monotherapy maintenance 2: HD-MTX plus ibrutinib 7: RTX-HD-MTX plus ibrutinib	560 to 840 mg	19.7 months (range, 12.7–27.1)
Mao 2018	China	Retrospective Study	3	N/A	High-dose methotrexate-based chemotherapy	Combined therapy	560 mg once daily	N/A
Soussain 2019	France	Phase II clinical trial	52	70 y (range, 52–81 y).	High-dose methotrexate-based chemotherapy	Monotherapy Steroids in initial four weeks for cerebral edema	560 mg once daily	25.7 months
Lewis 2019	Australia	Retrospective Study	8	65 y	High-dose methotrexate-based chemotherapy with radiotherapy, rituximab, and other chemotherapy. Median 1 (0–3) before treatment	Monotherapy in some patients and combined therapy (radiation plus chemotherapy) in the rest.	Daily dose was 560 mg (range 420–840 mg);	14 months
Lauer 2020	Germany	Retrospective Study	5	63 y (range: 53–82)	All patients were heavily pretreated (median of two prior treatment regimens), with 100% of patients receiving high-dose cytarabine and/or HD-MTX. Some received high-dose chemotherapy and autologous stem cell transplantation.	Monotherapy in some patients and combined therapy (radiation plus chemotherapy) in the rest.	560 mg once daily	427 days (range: 75–711)

## Data Availability

The datasets generated and/or analyzed during the current study are available from the corresponding author upon reasonable request.
